# Novel starting points for fragment-based drug design against human heat-shock protein 90 identified using crystallographic fragment screening

**DOI:** 10.1107/S2052252524012247

**Published:** 2025-01-17

**Authors:** Liqing Huang, Weiwei Wang, Zhimin Zhu, Qianhui Li, Minjun Li, Huan Zhou, Qin Xu, Wen Wen, Qisheng Wang, Feng Yu

**Affiliations:** a Shanghai Institute of Applied Physics, Chinese Academy of Sciences Shanghai201800 People’s Republic of China; b University of Chinese Academy of Sciences Beijing100049 People’s Republic of China; c Shanghai Synchrotron Radiation Facility Shanghai Advanced Research Institute, Chinese Academy of Sciences Shanghai201204 People’s Republic of China; University of Melbourne, Australia

**Keywords:** crystallographic fragment screening, fragment-based drug discovery, HSP90

## Abstract

Heat-shock protein 90 (HSP90) is a highly active molecular chaperone that plays a crucial role in cellular function. It facilitates the folding, assembly and stability of various oncogenic proteins, particularly kinases and transcription factors involved in regulating tumor growth and maintenance signaling pathways. Consequently, HSP90 inhibitors are being explored as drugs for cancer therapy. Crystallographic fragment screening is a novel screening method that has been developed in recent years for fragment-based drug discovery and is known for its high hit rate and its ability to provide direct insights into the complex structures of proteins and compounds. In this paper, high-diffraction-resolution crystals of the N-terminal domain of human HSP90α were employed in crystallographic fragment screening to discover binding fragments and binding sites. A diverse library of 800 structurally distinct fragments was screened, yielding 91 starting points for the fragment-based drug design of new HSP90α N-terminal inhibitors. Nearly a thousand crystals were measured, with 738 being processed and phased using a highly automated data-processing pipeline including data reduction and phasing, refinement and hit identification via *PanDDA* multi-data-set analysis. The 91 identified compounds bind to eight distinct regions of the HSP90α N-terminus, with 63 fragments located in the ATP-binding pocket and its surroundings, thus demonstrating the potential for the development of HSP90α- and ATP-binding inhibitors. This study emphasizes crystallographic fragment screening as a powerful method that can effectively identify fragment molecules and inhibitors that bind to HSP90α, contributing to ongoing efforts in cancer drug discovery.

## Abstract 2

In this first instance of crystallographic fragment screening completed by the crystallographic fragment-screening platform of the Shanghai Synchrotron Radiation Facility (SSRF) in China, 800 fragments were screened and 91 compounds were identified to bind at eight different sites.

## 1. Introduction

The heat-shock protein 90 (HSP90) family is a large family of heat-shock proteins with a molecular weight of approximately 90 kDa. HSP90 is one of the most active molecular chaperones in cells and is essential for the proper folding and activation of a large number of substrate proteins (Richter *et al.*, 2006 ▸; Ravagnan *et al.*, 2001 ▸). Currently, at least 280 HSP90 client proteins have been extensively studied, which include well known oncogenes, including several potent anticancer drug targets such as HER-2, BCR-Abl, VEGFR and EGFR (Ren *et al.*, 2014 ▸). HSP90 stabilizes various oncoproteins, including hypoxia-inducible factor 1 (HIF1), v-Src, human epidermal growth factor receptor 2 (ErbB2) and telomerase, and thus regulates several pathways that are dysregulated in cancer (Miyata *et al.*, 2013 ▸). Additionally, the more sensitive client proteins are typically those with unstable conformations that are involved in mutations leading to abnormal signaling in tumor cells. Among the most prominent HSP90 client proteins associated with cancer is the tumor suppressor gene p53, which is mutated in half of all cancer patients (Schulz-Heddergott *et al.*, 2018 ▸). Research has demonstrated that pharmacological inhibition of HSP90 leads to p53 degradation and significantly prolongs the survival of mice harboring mutant p53 (Alexandrova *et al.*, 2015 ▸). These findings suggest that inhibition of HSP90 represents a promising therapeutic strategy in cancer treatment. Structurally, HSP90 is a dimer composed of monomeric subunits that consist of three well defined domains: the N-terminal domain (NTD), the middle domain (MD) and the C-terminal domain (CTD) (Birbo *et al.*, 2021 ▸). The N-terminal domain is highly conserved in HSP90 homologs and contains an ATP-binding motif that belongs to the GHKL superfamily (Dutta & Inouye, 2000 ▸). This ATP-binding site is essential for the ATPase activity of HSP90, which is necessary for its functional cycle and for binding client proteins. The middle domain modulates ATPase activity by interacting with the γ-phosphate of ATP and features a large hydrophobic surface that facilitates the proper folding of client proteins. The C-terminal domain is responsible for two key functions: calmodulin binding and homodimerization (Whitesell & Lindquist, 2005 ▸; Lavery *et al.*, 2014 ▸). Interestingly, the C-terminal domain also possesses an ATP-binding site and acts as an allosteric regulator of the N-terminal ATPase activity. The functionality of HSP90 is dependent on the binding of ATP and the hydrolysis of ATP at the N-terminus (Sőti *et al.*, 2003 ▸).

The HSP90 protein, in the absence of ATP binding, forms a loose dimer through the CTD, known as the ‘open state’. After ATP binds to the NTD, the ATP lid (defined as residues Met98–Val136 including three helical segments) flips up and covers the binding pocket, leading to an overall structural rearrangement into a twisted and compact dimer known as the ‘closed state’. In the closed state, the NTD also participates in formation of the dimer, which is one of the reasons why the closed-state structure becomes more compact. The MD participates in the formation of the ATP hydrolysis center. After ATP hydrolysis, accompanied by the release of ADP and a phosphate group, the NTD homodimer dissociates and HSP90 returns to the open state. Several anticancer drugs, such as geldanamycin and radicicol, inhibit the activity of HSP90 by binding to the ATP-binding site of the HSP90 NTD, leading to the abnormal folding of HSP90 client proteins and inhibiting tumor growth (Supko *et al.*, 1995 ▸; Sydor *et al.*, 2006 ▸; Biebl & Buchner, 2019 ▸). Therefore, the ATP-binding site of the human HSP90α NTD has become a hot target for the development of antitumor drugs. There are over 300 crystal structures of the HSP90α NTD bound to different ligands in the Protein Data Bank, but there is currently no systematic study reporting on the adaptability of HSP90 to various small-molecule fragments.

In the past 20 years, fragment-based drug discovery (FBDD) has been extensively employed in the development of new chemical scaffold drugs (Erlanson, 2012 ▸). At least six drugs have successfully been marketed, and over 50 are currently undergoing clinical trials (Woodhead *et al.*, 2024 ▸). FBDD serves as an alternative method to high-throughput screening (HTS; Bissaro *et al.*, 2020 ▸). HTS involves the examination of large compound libraries containing thousands or even millions of compounds through biochemical or biophysical methods to identify potential drug candidates (Fox *et al.*, 2006 ▸). Although HTS is labor-intensive and costly, it can sample only a small fraction of the vast combinatorial chemical space. While HTS is highly effective in identifying compounds that bind tightly to target proteins, these molecules often exhibit poor drug-like properties due to high lipophilicity or unfavorable pharmacokinetic characteristics (Shun *et al.*, 2011 ▸). In contrast, FBDD explores smaller libraries containing hundreds to thousands of simple molecules (molecular weight < 300 Da, *c*log*P* < 3, hydrogen-bond donors or acceptors < 3; Congreve *et al.*, 2003 ▸). The lower molecular weight of these compounds allows a small compound library to effectively sample chemical space and may lead to the discovery of new binding sites that are challenging to identify with higher molecular weight compounds. Although the initial hit compounds may exhibit low affinity, their binding affinity can be further enhanced through fragment growth or fragment merging (Kirsch *et al.*, 2019 ▸). Crystallographic fragment screening is a novel FBDD method (Martin *et al.*, 2023 ▸) that has emerged in recent years. This approach boasts a high hit rate and can directly yield complex structures of proteins and compounds, facilitating subsequent compound design (Davies & Tickle, 2012 ▸). In this report, we present the results of a crystallographic fragment screen targeting the N-terminal domain of HSP90. A total of 800 compounds were screened, with 91 observed across eight distinct binding regions.

## 2. Materials and methods

### 2.1. Protein expression and purification

The N-terminal domain of HSP90 (residues 9–236) was induced in *Escherichia coli* BL21(DE3) cells using LB broth with 0.6 m*M* isopropyl β-d-1-thiogalactopyranoside. After 5 h of induction at 30°C, the bacteria were centrifuged at 7000*g* for 10 min. The resulting cell pellet was resuspended in lysis buffer *A* (20 m*M* Tris–HCl pH 7.5, 300 m*M* NaCl, 5 m*M* β-mercaptoethanol, 10% glycerol) and lysed using a high-pressure homogenizer. The lysate was then centrifuged at 30 000*g* for 30 min. The supernatant was loaded onto a 5 ml Ni–NTA column that had been pre-equilibrated with lysis buffer. The target protein was eluted using buffer *A* supplemented with 300 m*M* imidazole. Subsequently, the protein was loaded onto a Superdex 75 16/60 column that had been equilibrated with buffer *B* (20 m*M* Tris–HCl pH 7.5, 150 m*M* NaCl, 10% glycerol). Finally, the target protein was concentrated to 20 mg ml^−1^ using a 10 kDa molecular-weight cutoff ultrafiltration tube (Millipore). Aliquots were snap-frozen in liquid nitrogen and stored at −80°C until further experiments.

### 2.2. Protein crystallization and compound soaking

Initially, we grew HSP90α NTD (HSP90^N^) crystals using the previously reported crystallization conditions (Cao *et al.*, 2017 ▸). However, the crystals grown under these conditions exhibited poor tolerance to DMSO and other compounds, rendering them unsuitable for crystallographic fragment screening. We attempted to improve the tolerance of the crystals to DMSO/compounds by using different PEGs and increasing the concentration of PEG. When the precipitant was changed from 8% PEG 3350 to 22% PEG 4000, the size of the HSP90^N^ crystals decreased, but their tolerance to DMSO/compounds improved significantly, with no significant change in the crystal diffraction resolution. The final crystallization conditions were 100 m*M* Tris–HCl pH 8.5, 200 m*M* MgCl_2_, 22% PEG 4000 and a protein concentration of 20 mg ml^−1^. HSP90^N^ crystals were obtained by mixing 200 nl protein solution with an equal volume of reservoir solution in SWISSCI MRC-3 plates, followed by incubation at 4°C for 3–5 days.

A fragment library which contained 800 compounds (Topscience Biotech) was utilized for this screening campaign, with the compounds stored in DMSO at a concentration of 500 m*M*. A noncontact nanolitre acoustic pipette (ECHO650, Beckman) was employed to dispense the compounds into the crystallized droplets (Collins *et al.*, 2017 ▸). Each selected crystallization droplet received only one compound, resulting in a final concentration of 25 m*M*. The crystals were then incubated at 18°C for 6 h. Based on diffraction tests, cryoprotectant was deemed unnecessary. All crystals were directly harvested and rapidly cooled in liquid nitrogen.

### 2.3. Data collection

A total of 948 data sets encompassing 768 compounds were collected semi-automatically on BL02U1 (Liu *et al.*, 2023 ▸) and BL10U2 (Xu *et al.*, 2023 ▸) at the Shanghai Synchrotron Radiation Facility. Prior to data collection, sample information was imported into the data-collection system (Yu *et al.*, 2019 ▸, 2024 ▸). The samples can be mounted and centered automatically. The current sample information and data-storage path are also updated and generated automatically. Subsequently, manual confirmation is required before clicking to begin data collection. All crystals were measured with 360° rotation at 100 K using an EIGER2 S 9M detector or an EIGER X 16M detector.

### 2.4. Data processing and hit identification

Data reduction was performed using *Porpoise* (Yu *et al.*, 2019 ▸; Kabsch, 2010 ▸), *xia*2 (Winter, 2010 ▸) or *autoPROC* (Vonrhein *et al.*, 2011 ▸). After excluding low-quality data, a total of 745 data sets were utilized for further analysis, with each data set corresponding to a specific compound. These data sets belonged to space group *I*222, with diffraction to a mean resolution of 1.79 Å. The proportion of reflections assigned the free *R* flag defaults to 5% (Beilsten-Edmands *et al.*, 2020 ▸), and selections were made for each data set according to the default settings. The structure of the apoprotein was solved using *Phaser* (McCoy *et al.*, 2007 ▸) based on data from the best-diffracting apo crystal using PDB entry 3t0h as the search model. It was refined through cycles of iterative model building with *Coot* (Emsley *et al.*, 2010 ▸) and refinement with *Phenix* (Liebschner *et al.*, 2019 ▸). This structure was subsequently used as a model for the *Dimple* pipeline (https://ccp4.github.io/dimple/). The *Dimple* pipeline automatically selected Fourier synthesis or molecular replacement to determine the initial phase and employed *REFMAC* (Murshudov *et al.*, 2011 ▸) for structure refinement. A total of 738 refined data sets with an *R*_free_ of less than 40% were used in *PanDDA* (Pearce *et al.*, 2017 ▸) analysis to identify hit compounds.

## 3. Results

### 3.1. Crystal optimization and data collection

For successful crystallographic fragment screening, well diffracting crystals and reproducibility are required. At the same time, the appropriate concentrations of DMSO and compounds must also be tested to ensure that the crystals can still maintain ideal diffraction resolution and data quality after soaking. Consequently, various HSP90^N^ crystal hits and different concentrations of DMSO/compounds were tested and optimized. A total of 32 crystallization droplets were utilized to test the tolerance of crystals to DMSO/compounds. Compounds were added to the droplets to final concentrations of 25, 50, 75 and 100 m*M*, respectively, with corresponding DMSO concentrations of 5%, 10%, 15% and 20%. Changes in crystal stability were observed every 2 h over a 12 h period. It was observed that the crystals of HSP90^N^ could not tolerate compounds at concentrations as high as 75 and 100 m*M*. Diffraction experiments indicated that the data quality of the 25 m*M* compound-soaked crystals was superior to that of the 50 m*M* compound-soaked crystals.

**Figure 1:**
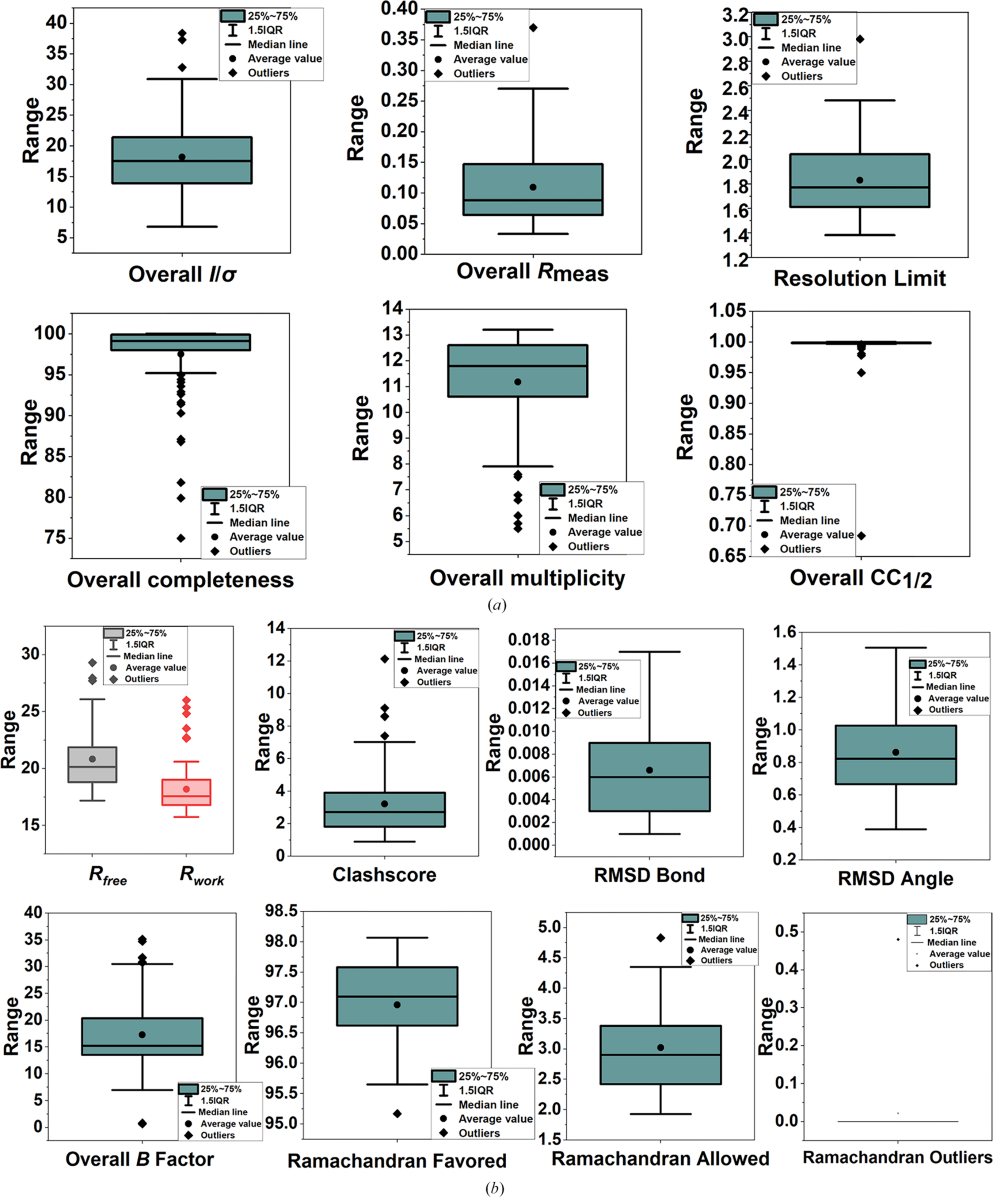
The distribution of data-reduction and structure-refinement statistics for all 91 data sets. (*a*) The range of key statistics for data collection. Overall *I*/σ, overall *R*_meas_ and resolution limit are relatively concentrated. In contrast, the distributions of overall completeness, overall multiplicity and overall CC_1/2_ are more dispersed, with a significant difference between the maximum and minimum values. (*b*) The range of some statistics for structural refinement. Although *R*_free_ and *R*_work_ have some outliers, their overall distribution is relatively concentrated. The difference between *R*_free_ and *R*_work_ is maintained within 5%. The distributions of overall *B* factor and clashscore are relatively wide, while other parameters are more concentrated. For the r.m.s.d.s of bond lengths and angles, there are no outliers in the distribution of these two data sets. All data for Ramachandran allowed, Ramachandran outliers and Ramachandran favored are concentrated. Notably, for the Ramachandran outliers most of the data are zero, which adversely affects the graphical representation.

Finally, the optimized crystals were fully grown after being incubated at 4°C for 3–5 days in crystallization buffer containing 22% PEG 4000. They remained stable after soaking for 6 h in crystallization droplets with added compounds at a final concentration of 25 m*M*. All data sets were collected using consistent parameters. A total of 948 data sets covering 768 compounds were obtained, of which 738 were utilized in *PanDDA* analysis to identify hit compounds. These crystals belonged to space group *I*222 and exhibited diffraction to an average resolution of 1.79 Å. All structures containing fragments were solved using the published structure of HSP90^N^ (PDB entry 3t0h; Li *et al.*, 2012 ▸) as a model for molecular replacement. Fig. 1 ▸ illustrates the statistical distribution of data reduction and structural refinement for all 91 data sets. Detailed data-reduction and refinement statistics are presented in the supporting information. One HSP90^N^ monomer was identified in the asymmetric unit. Electron density was observed for residues 6–310, with only five residues missing at the N-terminus and one residue missing at the C-terminus.

### 3.2. Hit compounds

**Figure 2:**
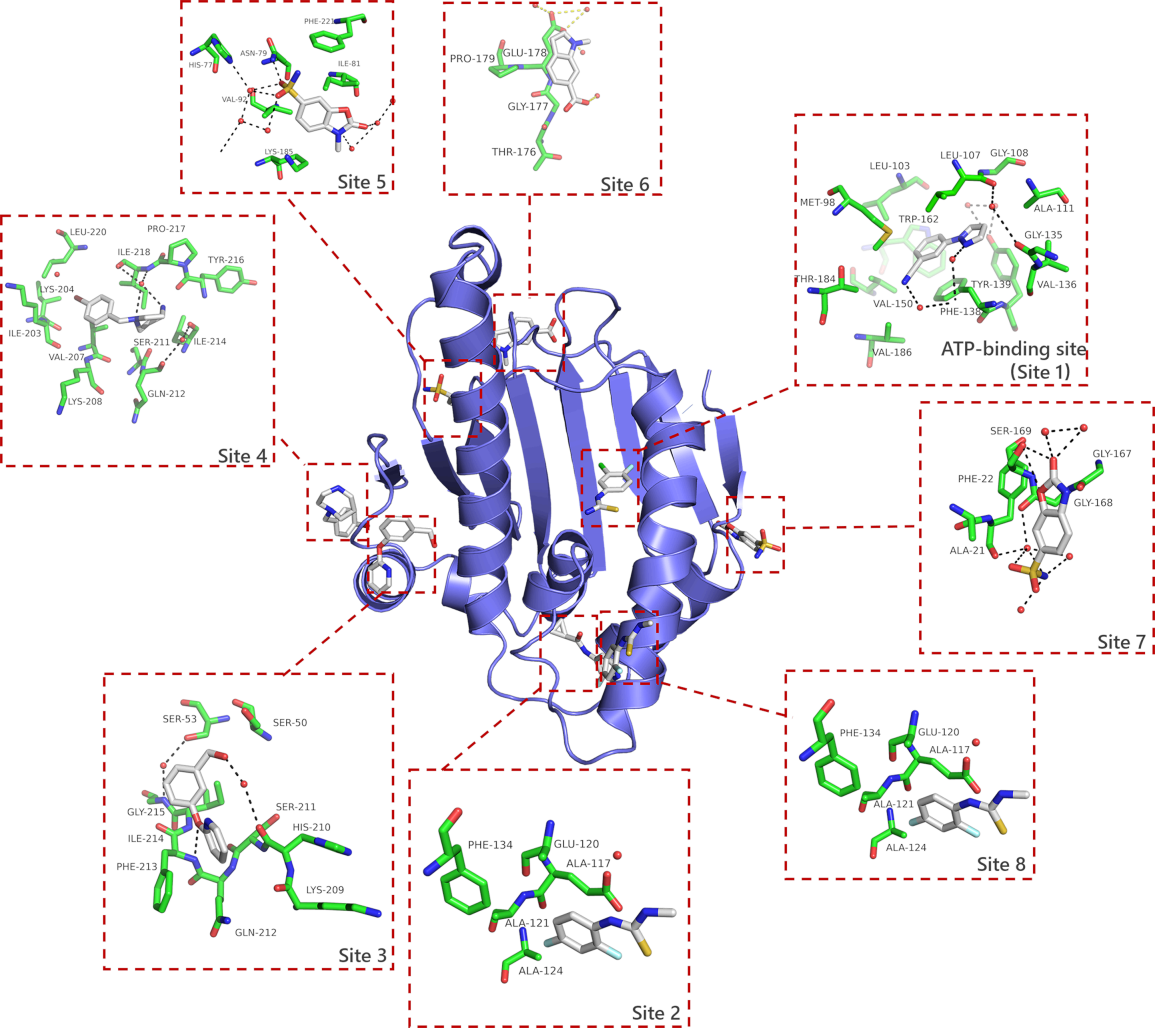
A total of 91 compounds bind to eight distinct regions of HSP90^N^. This figure illustrates the distribution of binding compounds and sites identified through analysis using *PanDDA*. In addition to the ATP-binding site, we have sequenced and mapped other sites. Site 2 refers to the region between Leu29–Tyr38 and Ala121–Asp127, and it is noteworthy that this site is in proximity to the ATP lid. Site 3 and site 4 are also relatively close; site 3 is located between Lys41–Leu70 and His210–Gly215, while site 4 is situated between Glu200 and Phe221. Site 5 and site 6 are similarly close, with site 5 located near Pro179–Lys185 and site 6 near Gly177–Pro179. Additionally, site 7 is located close to Gly167–Ser169, and there is also site 8 near Ala111–Ala124.

A total of 91 compounds were identified as binding to eight distinct positions on the protein (see Fig. 2 ▸ and supporting information), yielding a hit rate of 11.375%. The resolution range of the data sets for these compounds spans from 1.38 to 2.98 Å, with a median resolution of 1.78 Å. Interestingly, eight of the 91 bound compounds were observed to bind to more than one region, resulting in a total of 101 unique binding events (see Fig. 2 ▸ and Supplementary Fig. S1). Among these, 63 compounds specifically bound to the ATP-binding pocket of HSP90^N^, leading to 71 binding events. The remaining 28 fragments interacted with seven other regions, accounting for 30 binding events. Additionally, 17 compounds were found to bind to three different sites on the surface of HSP90^N^. These binding events represent potential starting points for fragment-based drug design, and their binding sites will be discussed in further detail below.

### 3.3. ATP-binding site (site 1)

**Figure 3:**
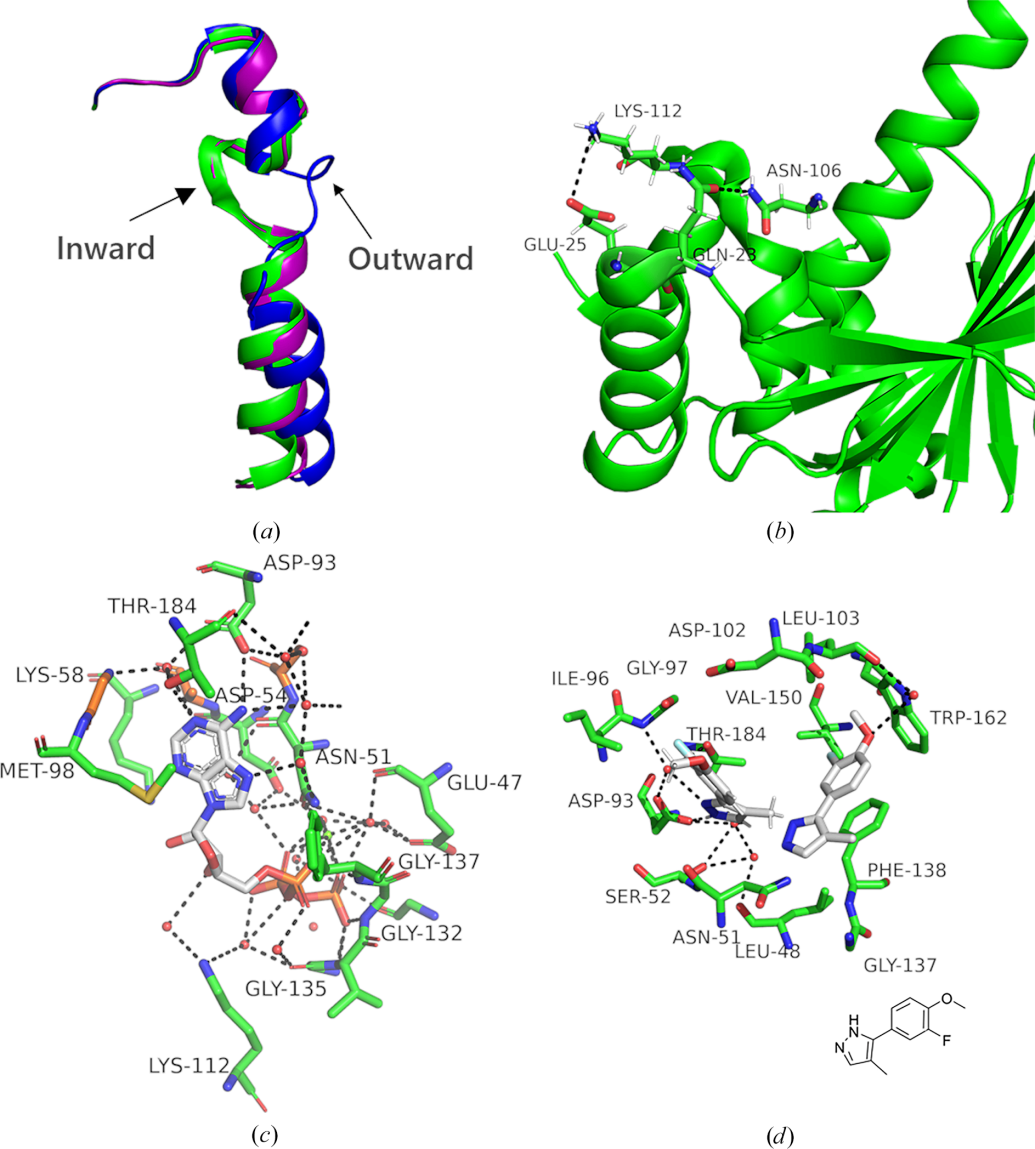
Overview of fragment binding in the ATP-binding site (site 1). (*a*) A schematic representation of three crystal structures, apo HSP90^N^, HSP90^N^–ATP and the binding fragment compound, illustrated in different colors (green for apo, blue for HSP90^N^–ATP and purple for the binding fragment). (*b*) A detailed display of hydrogen bonding in apo HSP90^N^ reveals hydrogen bonds between Glu25 and Lys112, as well as between Gln23 and Asn106. (*c*) An analysis of the HSP90^N^–ATP interaction shows that new hydrogen bonds are formed directly between Thr184 and Asp93 and ATP. Additionally, Lys112 forms two new hydrogen bonds to ATP, mediated by two water molecules. (*d*) A detailed interaction analysis between fragment 10T-0263 and HSP90^N^ indicates π–π stacking between the benzene ring of the fragment and the benzene ring of Phe138. There is a direct hydrogen-bonding interaction between the fragment and Trp162 and Asp93, along with water-mediated hydrogen bonding to Leu103, Thr184 and Gly97. Hydrogen bonds are represented as black dashed lines, while small red dots denote water molecules.

The most significant binding region is the ATP-binding site, which also constitutes the largest binding pocket. A total of 63 compounds are bound to the ATP-binding site, resulting in 71 binding events (see Supplementary Fig. S2). Previous studies utilizing the co-crystallization method to investigate the structure of the HSP90^N^ ATP-binding site, both with and without ATP, revealed that the region from Thr99 to Ala124 exhibits substantial conformational changes depending on the ATP-binding status. In the apo HSP90^N^ structure (PDB entry 3t0h), the segment from Thr99 to Ala124 forms a continuous helical pattern (Li *et al.*, 2012 ▸). However, the segment formed by Asn105–Ala111 does not conform to an α-helix; instead, it is more loosely structured and is oriented inwards towards the active pocket [see Fig. 3 ▸(*a*)]. In the Asp127–Leu143 helix, a β-turn (Gln133–Val136) is oriented inwards towards the active pocket. In the absence of ATP binding, hydrogen-bonding interactions occur between residues Glu25 and Lys112, as well as between residues Gln23 and Asn106 [see Fig. 3 ▸(*b*)].

ATP binds to the active site (PDB entry 3t0z), causing the Asn105–Ala111 segment to transition from a helical structure to a loop-like conformation, which is rotated 180° outwards from the pocket. Additionally, the β-turn formed by residues Asp127–Leu143 is modified to include only Phe134–Val136 (Li *et al.*, 2012 ▸). The original hydrogen bonds between residues Glu25 and Lys112, as well as between Gln23 and Asn106, are disrupted, leading to the formation of new interactions. Specifically, new hydrogen bonds are established between Thr184 and the N1 atom of ATP and between Asp93 and the N6 atom of ATP [see Fig. 3 ▸(*c*)].

In the case of fragment 10T-0263 [see Fig. 3 ▸(*d*)], there is π–π stacking between the benzene ring of the fragment and the benzene ring of Phe138. The O atom (O1) of the fragment forms hydrogen bonds to Trp162 and Leu103 through water-mediated interactions. Additionally, the N atom (N1) forms hydrogen bonds to Asp93, Thr184 and Gly97 via water molecules.

**Figure 4:**
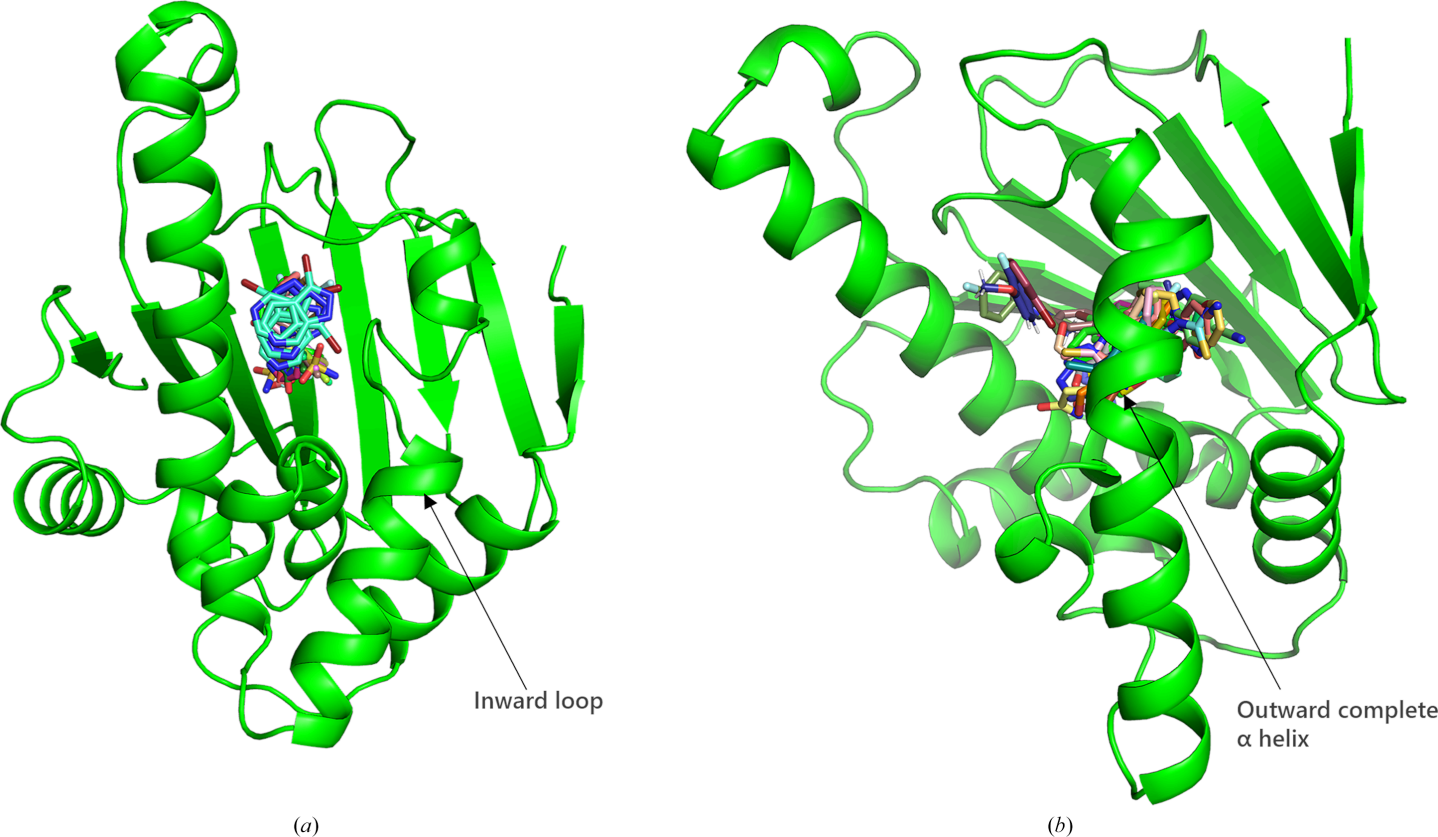
Two categories arise from the binding of fragments at the ATP-binding site. (*a*) An apo-like conformation is observed upon the binding of 19 fragments. None of the fragments interacted with ATP, and the Leu107–Val111 region forms a loop-like structure. (*b*) A distinct conformation, differing from that of ATP binding, is noted. In this case, 44 fragments stacked together like hairpins, and the Leu107–Gly114 segment formed a complete α-helix.

The ATP-activated pocket of HSP90^N^ comprises 38 residues (Prodromou *et al.*, 1997 ▸) and has a pocket volume of 784.9 Å^3^ (calculated using the *Proteins Plus* program). In this experiment, 63 fragments were bound within the pocket, resulting in 71 binding events that nearly filled the pocket. These 63 fragments can be categorized into two groups. 19 fragments bind similarly to the apo form, and their binding does not influence the conformation of the protein. The segment Ile104–Ile110 forms a loose loop [see Fig. 4 ▸(*a*)]. Analysis of the interactions between these fragments and the protein reveals that 15 fragments can form hydrogen bonds to Asp93, a key amino acid, either directly or through water mediation. The binding of the remaining 44 fragments exhibited a conformation distinct from that of ATP binding, resembling the conformation of PDB entry 1uy6 (Wright *et al.*, 2004 ▸). The primary alteration in this conformation was the formation of a complete helix by Asn105–Ala111. When bound in the active pocket, the 44 fragments collectively appear as a ‘clamp’ around Asn105–Ala111 [see Fig. 4 ▸(*b*)], while Lys100–Ala124 form a complete α-helix. The Asn105–Ala111 segment is ‘expanded’ due to the insertion of the fragment into the pocket. Further analysis revealed that 18 fragments exhibit π–π stacking interactions with Phe138, causing this segment of the helix to shift outward. The binding of these fragments also resulted in the formation of new hydrogen bonds, which could occur directly or through water-mediated interactions between these 44 fragments and Leu48, Asn51, Ser52, Asp93, Tyr139 and Thr184. The formation of these hydrogen bonds ‘pushes’ the fragments closer to Asn105–Ala111, leading to expansion of this helix.

### 3.4. A novel binding site near the ATP lid (site 2)

**Figure 5:**
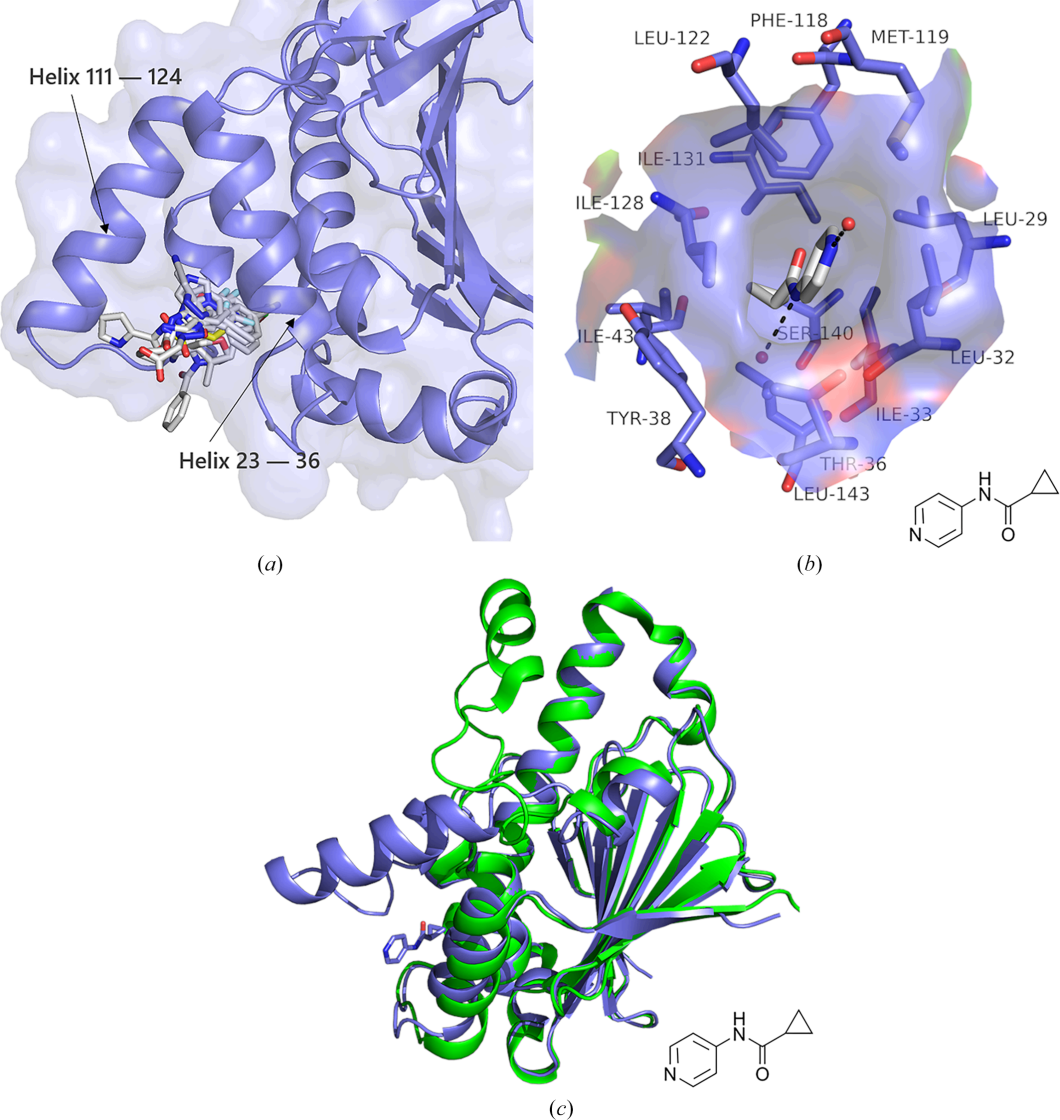
Fragment binding to site 2 adjacent to the ATP lid results in changes to the backbone. (*a*) The location of 13 clustered fragments is illustrated. A pocket is formed between helices 23–36 and 111–124. (*b*) Detailed interactions between the fragment and HSP90^N^ are depicted. Fragment 2X-5009 (gray) interacts solely with water through hydrogen bonds, yet is deeply embedded within this pocket. Hydrogen bonds are represented as black dashed lines, while small red dots indicate water molecules. (*c*) A comparison of the opening and closing of the ATP lid is shown. The blue color represents the open conformation following fragment binding to this site, while green (PDB entry 8b7i; Henot *et al.*, 2022 ▸) denotes the closed conformation.

A cluster of 13 fragments is situated in a pocket adjacent to the active site, with all binding fragments containing at least one aromatic ring that interacts with the surrounding polar side chains (Leu29–Tyr38 and Ala121–Asp127; see Fig. 5 ▸ and Supplementary Fig. S3). When a fragment binds to this pocket, the ATP lid adopts an open state. This stable open state can block ATP binding and disrupt the transition between the open and closed conformations of the HSP90 dimer, rendering this newly generated conformation incapable of binding to many key client proteins (Mimnaugh *et al.*, 1996 ▸; Johnson *et al.*, 2010 ▸; Biebl & Buchner, 2019 ▸).

### 3.5. Surface binding sites

In addition to the two primary binding sites mentioned above, we also identified six additional binding regions located on the surface of the protein.

**Figure 6:**
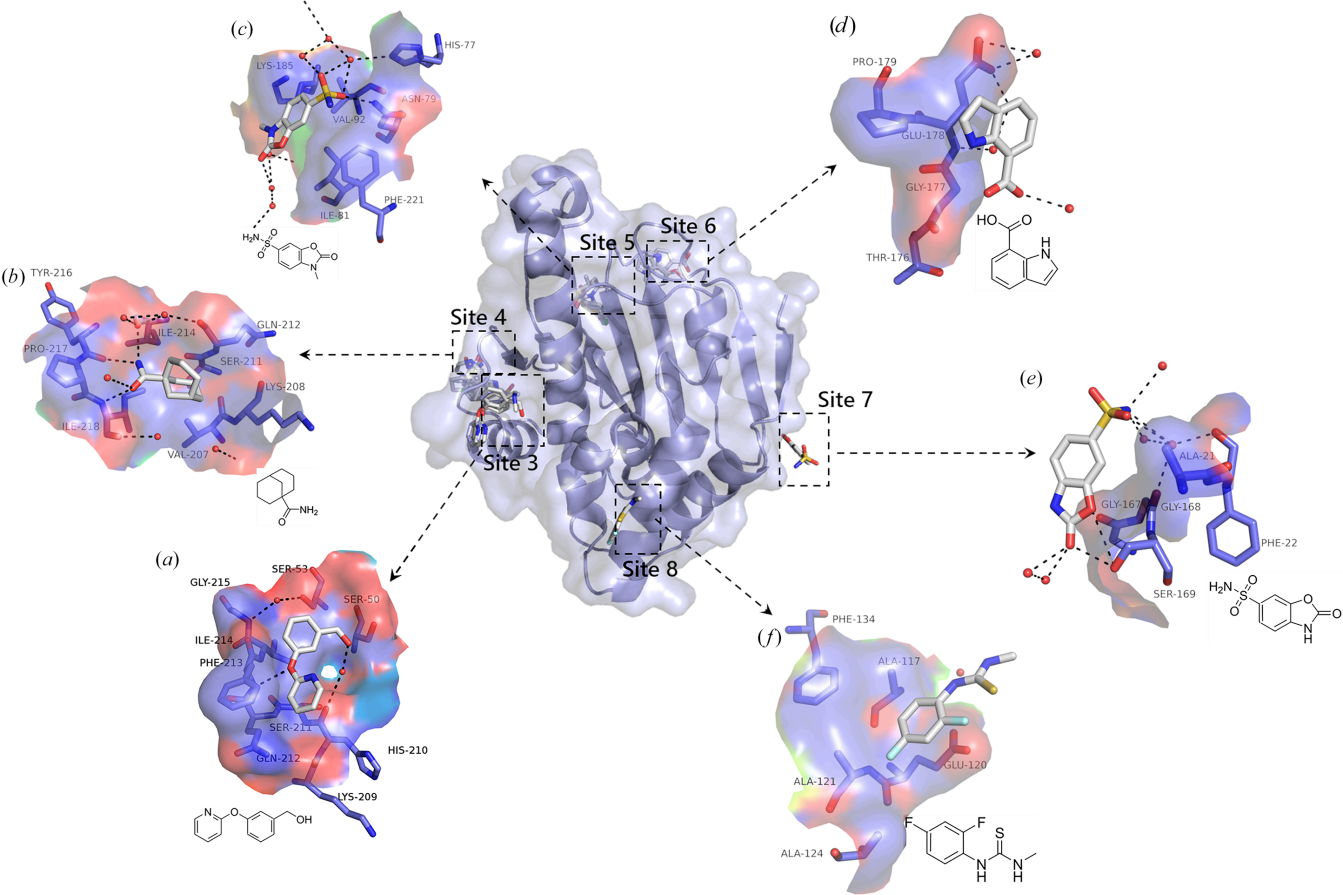
The profiles of six sites located on the surface of HSP90^N^ and their representative binding fragments are presented. (*a*) A close-up demonstration of the interaction between fragment Fr12938 and site 3 reveals a direct hydrogen bond to Phe213 and a water-mediated hydrogen bond to Ser211. (*b*) Fragment Fr14229, bound to site 4, forms a direct hydrogen bond to Ile218 and water-mediated hydrogen bonds to Ile214 and Ser211. (*c*) At site 5, fragment Fr13430 interacts with Asn79 through direct hydrogen bonding and with His77 via water-mediated hydrogen bonding. (*d*) At site 6, the primary interaction is hydrophobic rather than hydrogen bonding between the fragment and the amino acids. (*e*) Fragment Fr13229, when combined with site 7, results in a hydrogen-bond interaction with Ser169 and water-mediated hydrogen-bond interactions with Phe22 and Gly168. (*f*) Fragment Fr12961 binds to site 8 without significant interaction. (Hydrogen bonds are indicated by black dashed lines and small red dots represent water molecules.)

Binding site 3 is situated in the crevice between Lys41–Leu70 and His210–Gly215. There are a total of three compounds that bind in this region, each interacting with Gln212 and Phe213 through hydrogen bonding [see Fig. 6 ▸(*a*) and Supplementary Fig. S4]. Binding site 4 is located near Glu200–Phe221, where five compounds are found, each forming hydrogen-bond interactions with Lys204, Ile214, Tyr216 and Ile218 [see Fig. 6 ▸(*b*) and Supplementary Fig. S5]. Binding site 5 is located near Pro179–Lys185. There are a total of four compounds binding in this region, each interacting with Asn79 through hydrogen bonds [see Fig. 6 ▸(*c*) and Supplementary Fig. S6]. Previous literature has not reported any fragment binding in the aforementioned three regions. According to the available data in the PDB, in PDB entries 6cji and 3h80 only 1,2-ethylene glycol is bound near these three regions (Whitesell *et al.*, 2019 ▸). Binding site 6 is located near Gly177–Pro179. There is no direct hydrogen bonding between the three fragments in this region and HSP90^N^ [see Fig. 6 ▸(*d*) and Supplementary Fig. S7]. The fragments interact with protein mainly through hydrophobic interactions. In the reported structures, we found that in the structure of PDB entry 2vw5 (Zhang *et al.*, 2008 ▸) sulfate ion binds at this region. In addition, there are two other binding sites, each with one compound bound, located near Ser169 and Ala111–Ala124, respectively. Binding site 7 is located near Gly167–Ser169. Only one fragment binds to this region and forms hydrogen-bonding interactions with Ser169. Previous reports did not mention the combination of small molecules. According to the existing data in the PDB (PDB entry 4yky, unpublished work), there is glycerol bound near this area. There is a fragment that binds close to Ala111–Ala124 (site 8); the literature (PDB entry 1osf) reports the binding of acetic acid (Jez *et al.*, 2003 ▸) in proximity to this site. These regions have not been reported with fragments before, and the interaction between these fragments and the protein does not appear to be as strong. It is speculated that ‘collision’ or ‘sticking’ in this area may not have much effect on the change of function of HSP90^N^.

## 4. Discussion

HSP90 is a vital molecular chaperone that facilitates the proper folding and functioning of numerous proteins, including those associated with cancer. It stabilizes key cancer-related proteins such as p53, HER-2 and hypoxia-inducible factor 1 (HIF1), making it a significant target for cancer therapies (Ren *et al.*, 2014 ▸; Miyata *et al.*, 2013 ▸). Inhibitors that target the ATP-binding site in the N-terminal domain of HSP90 disrupt its function, leading to destabilization of its client proteins, which can inhibit tumor growth. In this study, crystallographic fragment screening was conducted on the N-terminal domain of HSP90 to identify potential small-molecule inhibitors. A library of 800 compounds was screened, and 91 were found to bind at eight distinct sites within the protein. This approach provides detailed structural information, which is essential for further optimization of these initial hits into more effective inhibitors through structure-based design.

In this study, we identified a novel binding pocket, referred to as binding site 2, located adjacent to helix 23–36 and helix 111–124. 13 fragments were found to bind to this pocket during our screening campaign. Binding site 2 is in close proximity to the ATP-binding site and has the potential to stabilize the open conformation of the ATP lid. We aim to optimize the interactions between the fragments and the pocket, and to integrate these findings with subsequent biochemical experiments to develop promising compounds and synthesize more stable and active derivatives. This approach may help in determining whether these compounds influence the function of HSP90.

Crystals for fragment screening require greater DMSO and compound tolerance compared with conventional crystallization experiments. Initially, we grew HSP90^N^ crystals using the crystallization conditions reported in the literature. Although the apo crystals exhibited good quality, only a limited number of crystals retained their original high diffraction quality after the addition of compounds. After several attempts, we modified the precipitant from PEG 3350 to PEG 4000 and increased the precipitant concentration from 8% to 22%. This adjustment significantly improved the tolerance of the crystals, allowing most of them to maintain sufficient diffraction quality for structural analysis even after the addition of compounds.

A total of 91 fragments from this crystallographic fragment screen resulted in 101 binding events distributed across eight distinct binding regions. This number of binding fragments corresponds to a scan of the HSP90^N^ binding pockets. By comparing these findings with the structures of small-molecule complexes of HSP90^N^ in the PDB, we identified nearly all known small-molecule binding pockets of HSP90^N^, including some pockets that are capable of binding only solvent molecules as documented in the PDB. In particular, site 2 between helix 23–36 and helix 111–124 is a novel binding pocket that accommodates a total of 13 fragments in this screen.

The affinity of the 61 fragments bound in the active pocket was evaluated using software. Notably, those fragments that are closer to Asn105–Ala111 contribute to the formation of a complete outwards helix at Lys100–Ala124. Furthermore, this specific conformation differs from the binding modes that are observed in apo HSP90^N^ and HSP90^N^–ATP. The top three scoring fragments were selected for design modifications aimed at enhancing their affinity. This approach will be complemented by biochemical assays, including molecular interaction mechanism analysis, cell-cycle analysis and apoptosis assays. The goal is to identify effective hit compounds.

Overall, this study successfully identified several new binding pockets in the HSP90 N-terminal domain through crystallographic fragment screening, including a novel site between helix 23–36 and helix 111–124 that stabilizes the open state of the ATP lid. Some fragments binding to the ATP-binding site can form a new conformation. A total of 91 fragments were discovered across eight distinct binding regions, providing valuable structural insights into potential drug targets. Optimization of these fragments could lead to the development of more potent and selective inhibitors. This research not only broadens our understanding of the binding pockets of the HSP90 N-terminus but also supports future structure-based drug discovery.

## Supplementary Material

PDB reference: N-terminus of HSP90α in complex with Fr12478, 7h9k

Supplementary figures. DOI: 10.1107/S2052252524012247/mah5001sup1.pdf

Ligand details. DOI: 10.1107/S2052252524012247/mah5001sup2.xlsx
