# Supplementary material for: Novel starting points for fragment-based drug design against human heat-shock protein 90 identified using crystallographic fragment screening
Source: IUCrJ. 2025 Jan 17;12(Pt 2):177–87. doi: 10.1107/S2052252524012247 (PMC11878448; doi:10.1107/S2052252524012247)
Supplement: Supplementary file 1 [file m-12-00177-sup1.pdf]

IUCrJ

Volume 12 (2025)

Supporting information for article:

Novel starting points for fragment-based drug design against human heat-shock protein 90 identified using crystallographic fragment screening

Liqing Huang, Weiwei Wang, Zhimin Zhu, Qianhui Li, Minjun Li, Huan Zhou, Qin Xu, Wen Wen, Qisheng Wang and Feng Yu

Figure S1 8 of these 91 bound compounds were found to bind to more than one region, leading to a total of 101 unique binding events. In particular, the fragment PS-4774, which results in 4 binding events in the ATP-binding pocket (Site1).

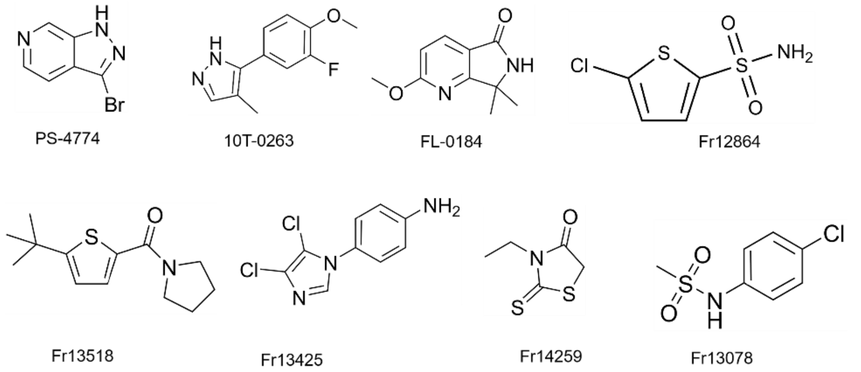

Figure S2 63 fragments observed binding in the ATP-binding site (Site1).

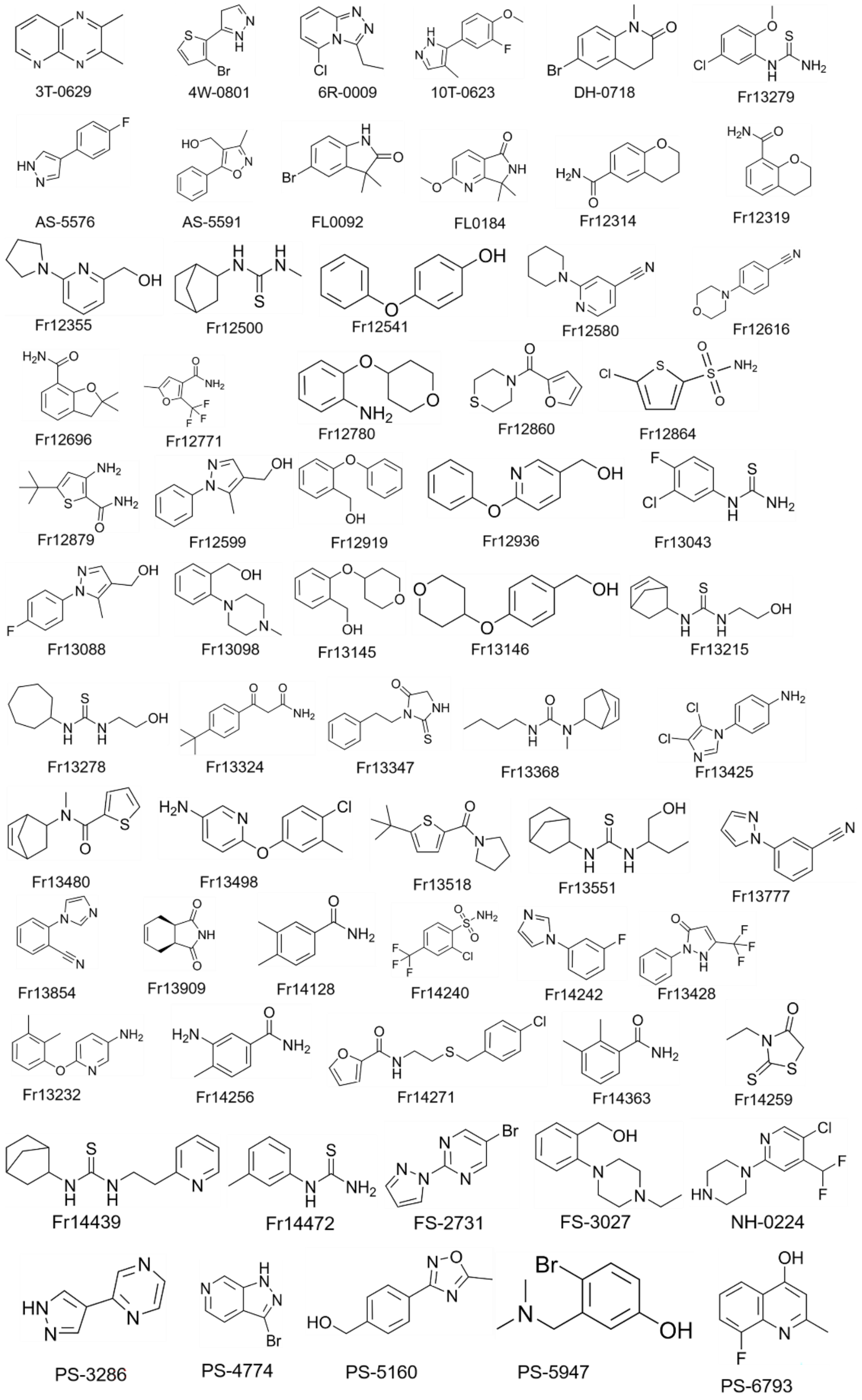

Figure S3 13 fragments observed binding in a novel binding Site 2 near ATP lid.

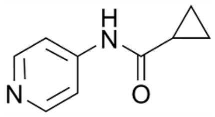

2X-5009

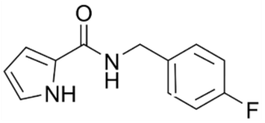

9R-0342

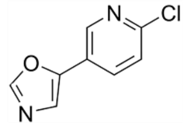

10X-0806

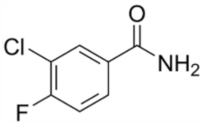

Fr12214

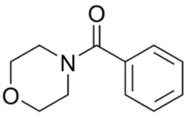

Fr12700

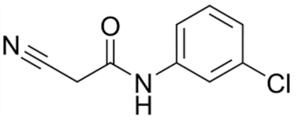

Fr12810

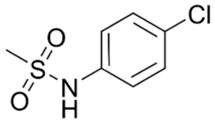

Fr13078

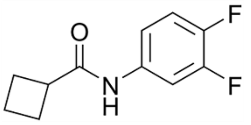

Fr13188

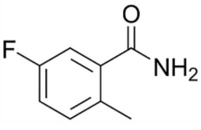

Fr13944

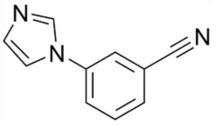

Fr14186

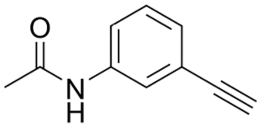

Fr14452

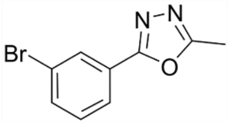

PS-5525

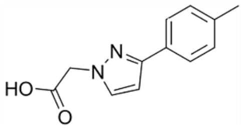

PS-3621

Figure S4 Three fragments bound in Site 3.

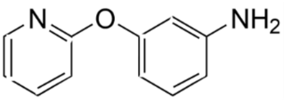

Fr12546

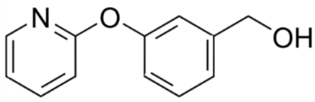

Fr12938

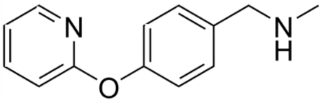

Fr13237

Figure S5 Five fragments bound in Site 4.

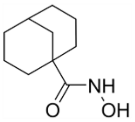

Fr12478

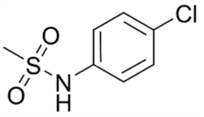

Fr13078

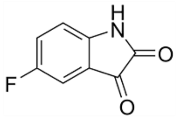

Fr13755

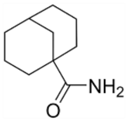

Fr14229

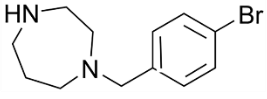

PS-3550

Figure S6 Four fragments bound in Site 5.

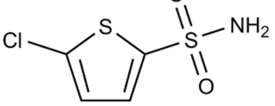

Fr12864

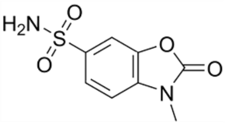

Fr13430

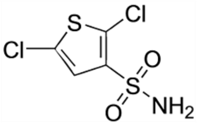

Fr13464

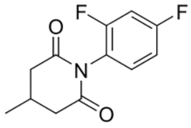

Fr13527

Figure S7 Three fragments bound in Site 6.

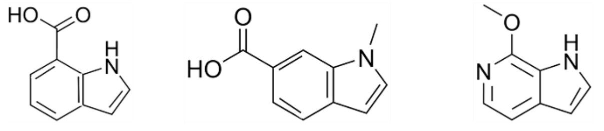

CC-0741 PS-4122 PS-4833
